# Eastern Australian Farmers Managing and Thinking Differently: Innovative Adaptation Cycles

**DOI:** 10.1007/s00267-023-01873-2

**Published:** 2023-09-05

**Authors:** David K. McKenzie, Janine Joyce, Kerstin K. Zander, Penelope A. S. Wurm, Kim M. Caudwell

**Affiliations:** 1 Faculty of Health, Charles Darwin University, Casuarina, NT Australia; 2 Centre for People, Place and Planet, Edith Cowan University, Joondalup, WA Australia; 3 Northern Institute, Charles Darwin University, Casuarina, NT Australia; 4 Research Institute Environment Livelihoods, Charles Darwin University, Casuarina, NT Australia

**Keywords:** Australian farmers, Climate-adaptation, Managing preparedness, Business continuity, Transformative farming, Resilient farming systems

## Abstract

The uncertainty of climate change is a significant challenge prompting Australian farmers to create different thinking and different management systems that ensure sustained farm business viability and continuity, particularly in extreme environments. The purpose of this study was to explore the conditions and adaptive processes for managing farm resilience and cyclic adaptation pathways, in response to climate change. A positive deviance sample of farmers was interviewed, and data was collected from a cohort of twenty-two climate change innovators across Eastern Australia. Grounded theory analysis of data identified three processes and two transactional maps of climate change adaptation, in this under studied farmer cohort. The development of the transactional maps found the resilience and preparedness processes as adaptive learning responses to the stressors of climate change. The processes of managing the business and resources were identified as markers of preparedness and resilience that ensured business viability and continuity. Farmers prepared for climate change through transforming make-over processes as an adaptive learning response to climate challenges. Mapping the cycle of adaptation identified the processes of socio-cognitive agency, learning from feedback and consequences, and contextual variables as critical elements of adaptation. The intervening socio-ecological processes of intelligence gathering and influencing, and socio-cognitive precursors, were found to regulate the adaptation cycle. The cycle was found to have both incremental and transformative transmission processes, and intervening processes of climate and contextual variables. The changing patterns and extremes of climate change were found to impact the growing season, and its potential, as unique variables that demand farm adaptation. Ultimately, this study identified potential points of influence for leveraging preparedness behaviours.

## Introduction

The impacts of climate change are causing significant social, economic and ecological disruptions to global systems of farming and food production (IPCC 2022). These climate changes challenges are prompting the need for well prepared and resilient farming and food systems that are adaptive and minimise risks of global food insecurity (Chriest and Niles 2018; Janssens et al. 2020). The development of farm resilience and preparedness requires transformative thinking and adaptive management to facilitate the behaviour change needed to transform farm systems (Colloff et al. 2021; Pahl-Wostl 2006; Park et al. 2012). The viability and continuity of Australian farming businesses depends on the transformative processes to prepare resilient farming systems that minimise the risk of climate change (Rickards and Howden 2012).

Australian farmers co-exist with the beneficial and adverse consequences of the natural environment as they interact with natural meteorological processes (Renaud et al. 2010). This necessitates a human environmental systems (HES) approach to building systems capability and reconciling accelerating risk arising from climate change, landscape, and socio-economic interactions (Scholz et al. 2011b). These interactions potentially amplify the human, economic and landscape costs and risks of future adverse consequences (Mozumder et al. 2009). Risk is the likelihood of the benefits of farming in certain geo-climatic locations as a proportion of the costs arising from exposure to adverse climate consequences (Paton and Buergelt 2017). Without managed adaptation the costs of farming in certain geo-climatic environments may exceed the likelihood of benefits. Therefore, unless farmers are ready to build resilience, and adaptively manage these risks, the viable continuity of farming in these locations may not be sustained.

Building farm resilience is an important precursor for how well farming systems in Australia adapt to the demands of climate change stressors (Asghari et al. 2021; Bardsley et al. 2018). The model of stress resistance and resilience over time accounts for these co-occurring adaptation and resilience processes (Norris et al. 2008). This model suggests that farms with more management capability and resource sufficiency will self-organise and adapt, whilst restoring systems from post-stressor states of transient dysfunction (i.e., climate disruption). By studying cohorts of farmers that have learnt to prepare resilient systems by adapting to situational and extreme climate stressors, knowledge is constructed of how these farmers think, prioritise, and strategically manage these as learning processes (Gorddard et al. 2016; Paton and Buergelt 2019).

Climate and environmental science have developed models that accounts for adaptation to the external conditions of climate change, as pathway processes of *action*-*learning-decision cycles* (Wise et al. 2014). The development of these models has applications of generalising descriptions of the phenomenon and specifying the elements and conditions that influence the processes. The Adaptation Action Cycles model of adaptation reflects how farmers interchangeably use *incremental* and *transformation* processes to adapt to the stressors of climate change (Park et al. 2012). Park et al. (2012) recognised that the *incremental* and *transformative* adaptation of farm systems were based on learning and cognitive processes. Farming systems were improved by thinking strategically, reframing, and questioning assumptions. Whereas, farms were *transformed*, by altering worldviews and structural context leading to a *different* system (Park et al. 2012). The reframing and normalisation of *incremental* and *transformative* as *better* and *different* were also important symbolic learning processes for changing the behaviours of disaster preparedness (O’Connell et al. 2020). The Transformative Adaptation Research Alliance (TARA) research framework further elaborated adaptation models by recognising the efficacy of contextual variables in *transforming* human systems (Colloff et al. 2017). Furthermore, the HES framework incorporated triple loop learning processes within a multi-level systems context (Pahl-Wostl 2009; Scholz et al. 2011a). This framework may better explain decision processes of adaptation within a systems context. This evolving conceptual understanding of adaptation necessitates further research of elements and processes that transmit and regulate adaptation and integrates models of resilience and preparedness processes.

Farm preparedness refers to adaptive adjustments that give farming systems protective agency and resilience to effectively respond to changing patterns and extremes events of climate change (Paton et al. 2014). The efficacy of business continuity as a preparedness management strategy had limited exposure in farm (James 2020), and disaster preparedness (Sawalha 2021) literature. Some exemplary Australian farmers are building farm resilience and developing systems of adaptive management the integrate *differen*t and *better* adaptive practices (Bardsley et al. 2018). Pahl-Wostl (2006) defines adaptive management as on-going systematic processes of learning, refining and reorganising management processes, strategies and practices. For instance, Bardsley et al. (2018) identified adaptive management and innovation as contributing to farm viability and business continuity. Lockwood et al. (2015) and Raymond et al. (2015) recognised that farm management competencies and change orientation were essential adaptation capacities. Literature on CCA has developed a generalised understanding of adaptive capacities in exemplar farmers. These farmers exhibit well-connected farmer informational networks, high degrees of environmental awareness, local knowledge, and strategic thinking and planning management capabilities (Marshall et al. 2016; Marshall et al. 2012).

The role of financial resources and preparedness behaviours was underrepresented in CCA research.,

The purpose of this study was to explore the farm management processes of resilience, and preparedness as an adaptive learning response to the stressors of climate change in an understudied farmer cohort. The study informs processes of adaptive farm management and resilience building that ensures business viability and continuity and reduced risk. The development of the transactional maps extends the theoretical understanding of resilience, transformation, and behavioural learning processes and factors that transmit and regulate farm adaptation. The maps have potential for identifying points of influence for leveraging farm resilience building and strengthening farmer preparedness.

## Methods

### Design

This qualitative priority study was part of a multiphase mixed methods program of research. The purpose of this study was to answer what/ how research question and generate theories (i.e., enhancement), and examine exemplar cases (i.e., initiation) (Creamer 2018). We applied the Human Environmental Systems multi-level framework (Scholz et al. 2011a), the functional and constructivist interpretive lens (Creswell and Poth 2017), and grounded theory methodology (GT) (Corbin and Strauss 2015) to data collection and analysis. Ethics approval for this study was obtained through the Human Research Ethics Committee at Charles Darwin University (H19096). The data was collected from farmer interviews and artifact documents of specific farmer interviews retrieved from a media and organisational websites, according to GT iterative and theoretical sampling, and analysis processes (Corbin and Strauss 2015).

### Sampling and Recruitment

The sample frame consisted of farmers in the eastern states of Australia (i.e., NSW, Victoria, SA, Queensland). The qualitative purposive approach was used to sample a cohort of farmers using positive deviance criteria (Pascale et al. 2010). The selection criteria for this cohort were that farmers had transformed their farming systems as a learning and behavioural response to anticipated and actual stressors of extreme climatic conditions. Participants were initially recruited by the executive officer of Farmers for Climate Action, in accordance with the positive deviance criteria. Subsequent participants were recruited via a snowball technique as a means of theoretical sampling. The theoretical sampling selected participants from various farm sectors, climatic zones, and specific climate extremes with purpose of further developing emerging GT concept and category codes.

### Participants and Collection

Twenty two participants were interviewed, consisting of 16 men and 6 women. All participants were located in eastern Australian states and represented diverse mixed cropping and livestock farm sectors (See Table 1).

**Table 1:** Participant demographics

State		Farm sector		Age		Gender	
NSW	10	Sheep	1	20–30	0	Women	6
Victoria	4	Cattle	7	30–40	0	Men	16
Queensland	3	Sheep-cattle	1	40–50	7		
South Australia	5	Crop-sheep	7	50–60	11		
		Crop	4	60–70	3		
		Dairy	1	70–80	1		
		Sugar	1				
Total	22		22		22		22

All participants were interviewed between March 2020 and June 2021. On-farm interviews and data collection were paused between April 2020 and April 2021 due to the COVID-19 restrictions and recommenced in May 2021. During the COVID-19 pandemic restrictions, interviews were conducted via telephone and the Zoom web-platform. Participants interviews were guided by the episodic interview technique (Flick 2009) and by a question guide that outlined the direction of the interview. The interview questions had focus on participant experiences of climate change and interactions with the landscape, business, production, and humans. The interviews lasted an average of 90 min and were audio recorded. The recordings from interviews were transcribed in accordance with the transcription protocol. Secondary situational maps of the farming systems and memo data was generated and included in the data analysis (Clarke 2003; Corbin and Strauss 2015).

### Data Analysis

The Corbin and Strauss (2015) procedures were used to analyse the data and build the theoretical model. The ATLAS.ti 9 (Evers and Silver 2014) data analysis software was used for data management and tracking the frequency that concepts were referred to in the data (references) as an indicator of concept importance. The data was systematically analysed using a three-step iterative coding scheme of open, axial, and selective coding (Corbin and Strauss 2015), in conjunction with constant comparison, theoretical sampling, and memo writing. The theoretical sampling was an emergent process of collecting different forms of data (e.g., situational maps, memos) and data from farmers exposed to challenging conditions (i.e., climate change) and across different farm sectors. Corbin and Strauss (2015) contend that challenging conditions are more likely to reveal adaptive processes embedded in the data. Data collection was finalised at the point of saturation, the point of data sufficiency with no new concepts emerging.

In the open coding process the interview transcripts, artefact documents, memos, and situational maps were conceptually labelled. Then, the emerging concepts were constantly compared for similarities and differences and integrated into higher order concept groups (i.e., tentative categories). The higher order concept groups of *better* and *different* forms of adaptation were coded in accordance with the composite set of criteria derived from Triple loop learning (Pahl-Wostl 2009) and Park et al. (2012), that distinguished *incremental* (i.e., *better*) as systems improvement, and *transformative* (i.e., *different*) as systems change. The analysis identified adaptation levers as influential factors that contribute or facilitate farmer adaptation to perceived or actual climate change by adoption of *better* and *different* thinking, behaviour, management regimes, practices, and strategies.

The data was organised via concept groups causal, situational, and intervening conditions, and strategies (action-interaction), and consequences. Situational maps were constructed from the analysis as shown in Fig. 3 to illustrate the context conditions. Clarke (2003) argues that situational maps act as units of analysis and better represents the actors, actants and discursive elements of the situational context than the Corbin and Strauss (1990) contextual matrix. The twofold axial coding process identified hierarchical category components of sub-category and respective properties and dimensions. Next, the various categories were assigned to the scheme groups and linkages assigned according to the emergent relationship (e.g., transmitting, regulating, interacting). Selective coding was final coding procedure of defining and refining the specifics of each category and creating explanations for respective categories that reflected the data. Lastly, models were created that reflected the elements and relationships of the adaptation processes.

## Results

Grounded Theory analysis yielded two core categories and three sets of adaptation processes. The core categories were ‘Dancing with uncertainty’ and ‘Sustaining viable continuity’. The processes were (1) framing changing climate uncertainty, (2) managing farm resilience, and (3) pathways of farmer adaptation. The processes led to the development of two transactional maps (i) Transactional maps of farm continuity; and (ii) Transactional map of cyclic adaptation.

### Process 1: Framing the Effects of Climate Change

This section explores how this cohort of interviewees from diverse geo-locations and farm sectors have common experiences of changes to windows and potential for plant and animal growth that result from changes in climate phenomena. The findings in Table 2 shows the categories, subcategories and sets of properties of ‘Changing climate uncertainty’ category as ‘Shifting variability patterns and ‘Emerging variability extremes’. These shifting climate patterns and extremes were framed by interviewees as the effects on growing windows and the potential for productive output (i.e., ‘Growing potential’). The elements of climate, and competition (i.e., weeds, pathogens), and management strategy variables contributed to the growing potential of crops and animals within the farming system. The findings in Fig. 3, maps these elements and their interactions with the elements of the farming system.

**Table 2:** Changing climate effects on growth windows and potential: categories and subcategories

Categories	Subcategories	Properties
Changing climate uncertainty	Shifting variability patterns	Changing distribution
		Changing seasonal amounts
		Changing reliability
	Emerging variability extremes	Intensifying climate conditions
		Creating hazard impacts
Shifting growing windows	Growing potential hazards	
	Growing season patterns	
	Locating growing geo-climate	
Creating growing potential	Growing potential competition	
	Manipulating growing strategies	

#### Effects of shifting climate patterns

Interviewees identified emerging shifts in variability patterns of rainfall and temperature that were both extreme and unprecedented:

*We had 42 degrees… it’s not a dry heat here…this wasn’t high humidity… we had a 70-knot westerly blowing for three days … I’ve never seen that before… we didn’t have any rain through those three years of winter…That’s never happened before… we had two winters without any frost that’s never happened before. These are subtle. (Cattle farmer, CE Qld)*.

Alongside these extreme unprecedented events interviewees described a regular truncated growing season due to rainfall switching-off during the critical plant growing phase:

*Our biggest growing period is October, but what’s been happening is that the rain’s been cutting out in September. (Cattle farmer, NE Vic.)*

In response, interviewees identified processes for managing and adapting to the non-growing season rainfall episodes that occurred as intense hot dry season rainfall events outside the usual growing window.

*Get massive dumps of rain that last for one month, then it will leave us for 4 to 5 months at a time, so we’ve basically set our grazing business up to harvest that rainfall event. (Mixed farmer, CW NSW)*.

#### Impacts of emerging variability extremes

The Emerging variability extremes subcategory was highly represented by droughts, heatwaves, floods, and bushfire events. Interviewees recounted the intensity of life-threatening impacts of large scale, out of control, cropping zone fires:

*[fire storm] …Like a really big round bale just rolling towards his house… it was making an awful noise… when this thing hit the house, the house just exploded…So if you were in the wrong place at the wrong time. (Cropping district, SA)*.

Alongside this were the increasing frequency and severity of heatwaves:

*The number of hot days in Northern Victoria over 42 degrees…1980s there was one day and the nineties…about five days… early two thousands it was 10 days…2010 to 2020, it was up to 16 to 25 days in heading towards forty. (Dairy farmer, Vic.)*.

Interviewees described the life threating flooding impacts of fast storm occurrences:

*I’ve noticed with our creek… floods are getting higher over time…had a storm here that lasted for 14* *h…we’re breaking records over time….more extreme events as time goes on … and the water came up into the second story….15* *min to get out of the place. It eclipsed the last record in 1990 by 4.6 metres…totally engulfed our sons house, I was a bit in denial… It’s just unbelievable. (Cattle farmer, Queensland)*

Consequently, interviewees view the effects on the land of flooding and rising sea-levels as farm viability hazard risks:

*[rainfall]… events where we didn’t really get a big flood…water stayed there for fourteen days…the ability to drain the property in a hurry is crucial. That’s, the main hazard we’re dealing with…we’ve got to deal with the flooding…biggest risk factor is sea level rise. (Sugar-cane farmer, N NSW)*

### Process 2: Managing Farm Resilience

This section explores the management of adaptation and resilience processes, as responses to the changing climate that ensure the viability and continuity of the farm. These interviewees continually repositioned the farm system as a reciprocal management response to the uncertain climate, as reflected by the ‘Dancing with uncertainty’ core category. The second core category of ‘Sustaining viable continuity’ reflects that interviewees sustain the continuity and viability of the farming landscape, family social identity, and business in the context of climate change challenges. The findings in Table 3 outlines the ‘Managing business directions’ and ‘Building resource capability’ categories, and subcategories that represent the management and resource adaptation processes.

**Table 3:** Managing adaptation and resilience: categories and subcategories

Categories	Subcategories
Managing business directions	Building resource capability
Managing business strategy	Building human capital
Managing farm transformation	Building financial resources
Managing farm efficiency	Developing capital resources
	Sustaining operating resources

#### Managing business directions

The category of ‘Managing business directions’ shows that interviewees are strategically managing the direction and position of the business system to accommodate social, macro-economic, and climate risk uncertainty. Strategic management regulated the recasting of the farming system through the agency of adopting *better* and *different* forms of adaptation.

##### Managing business strategy

Interviewees identified the strategic value of creating a business model that accommodated the localised adaptive demands of the changing climate:

*Modelling of my farm is done to get water [heavy rainfall episodes] off and, in the dry times retain moisture. (Sugar-cane farmer NNSW)*.

Interviewees described the rationale for creating a *different* and simplified business model:

*‘I wanted to go back to a simplified farming and not have the complexity of rations and feed systems and irrigation and heat in Northern Victoria [relocated to Southern Victoria]’ (Dairy farmer, Victoria)*

Consequently, interviewees preserved the core business in times of drought extremes to sustain the continuity of the farm:

*Like a formula one racing car driver, … make a bad decision…miss a corner, you can tumble…still be safe and sound in your shell…. parts of your business that you can drop off, …. and you’re going to be there to rebuild … plugin options to go on…business model that fits in with climate variability that matches stocking rate to carrying capacity. (Sheep-cattle farmer, SW NSW)*.

Interviewees identified the strategic necessity of planning adaptive business and operational readiness to accommodate the changing rainfall distribution patterns:

*Restructured business around that rainfall uncertainty, rather than planning around traditional wet seasons…success planning around individual rainfall events regardless of the time of the year they come*. *(Mixed farmer CW NSW)*

Interviewees planned and managed livestock resource systems to accommodate extreme climate events:

*[planned] business within our core breeding stock…25 to 30% of our total stock, because if its anymore than that [in dry years], we’re going to have to sell our core breeders … got to map out all these options and these plug in options to rebuild up to a hundred percent or 120%, depending on the season. (Sheep-cattle farmer, SW NSW)*.

Consequently, the platform of cloud-based software enabled monitoring and tactical planning in sustaining the dynamic balance between landscape carrying capacity and stocking rate:

*We use an outside consultant…who provides some input through MaiaGrazing, by using a cloud based product…cause we have just bought some stock…so we put together a feed budget to work out if we might have excess. (Sheep-cattle farmer, SW NSW)*.

Interviewees identified the necessity of managing risk to protect business viability against the increasing uncertainty of climate variability:

*How do you manage what everybody calls risk, I call variability…things aren’t always good, or they aren’t always bad, they’re somewhere in between, the climate component of that fits in there really well. (Grain farmer, SA)*.

Consequently, business strategies were packaged as adaptive levers for managing continuity risks. Strategies ranged from geographic relocation and property spread to enterprise diversification, altering scale, and flexible substitution:

*[climate change] increased the risk obviously… put out lot of money with cropping…we reduced the cropping, reduced back 50% or less, and the sheep which are more consistent….now that they’re worth something. (Mixed farmer, SE NSW)*.

Interviewees recasted systems of management to accommodate the integration of *different* strategic approaches. Consequently, well organised management systems were an adaptive lever and incorporated effective sets of strategies that led to cohesive performance overtime:

*When you get summer storms, which we’re getting more of, so we’re trying to keep as much stubble as we can [minimise run-off]…graze our stubbles briefly…if there’s not enough pasture cover, we often lock them up [sheep]…try and keep 70% ground cover. We cut silage in the good years and stick underground, so we feed that out to sheep in confinement yards with our silage heaps next door…use the silage to feed the ewes…gives more room for the lambs on the lucerne. (Mixed farmer, SE NSW)*.

##### Managing farm transformation

Interviewees identified variants of managing farm transformations with make-over processes, aimed at strengthening farm resilience and future preparedness. The farm make-over represents the purposeful creation of *better* and *different* farm structures, business and production functions, and management processes. These changes were accomplished through cycles of adaptation and learning that varied in complexity, time, and degree of systems change.

Interviewees described singular makeovers as one-time change, such as developing *different* bred types and sheep husbandry management that created a *different* flock genotype and *better* wool quality and ease of management:

*Move to something that’s shorn twice a year to manage the vegetation issues…there is less dust in them and less vegetable matter… don’t need to treat for lice or fly..they just don’t get into them with that skin type. (Sheep farmer, Riverina NSW)*

Interviewees described other make-overs as an evolving series of improvments to the system that together created the step of forming a *different* system:

*Cropping systems evolved by packaging strategies such as optimising ground cover, summer weed control, soil moisture testing, calendar based early planting, press wheel creating good seed soil contact and water harvesting contours, and varieties with temperature sensitive flowering. (Cropping farmers, SA)*

Interviewees commonly used project based make-overs in the form of self-contained developments. Projects, such as building livestock containments, structurally created *different systems*. Consequently*, different* management regimes led to *better* protective ground cover:

*We’ve invested in drought lots…got three of those confinements, so we can nearly lock up all our ewes and confine them for short periods of time…if there’s not enough cover. (Mixed farmer, SE NSW)*.

Some interviewees devised makeovers as series of sequential projects that evolved overtime:

*‘I’ve gone from trees [tree corridors] to sheep [different genetics- breed type] restablishing perennial pastures [different grazing management]. I tend to get really focused on one thing…so now I think probably soil is probably going to be my thing… tend to focus on things, ‘get that going, get that going’ and meanwhile everything else is going, keeps going’ (Sheep farmer, Riverina NSW)*

Other interviewees devised concurrent make-over projects to create a different farming system. These complex projects simultaneously created *different* landscape and infrastructure structural elements. Consequently*, different* management regimes and productivity functions led to *better* landcape and productivity outputs:

*We refenced the property…from two and a half thousand acres in a paddock …refenced those into five or six paddocks. There was 30,000* *acres…massive electric fencing and rewatering…did trial [large mob rotational grazing system]…realized that what we were seeing was working perfectly’ (Sheep-cattle farmer SE NSW)*

One interviewee described the bold move of creating a *different* farming system by relocating the farm business and operations to another geographical location. Consequently, risks of heat stress risk and irrigation water allocation shortfalls were averted:

*Crystal clear in my mind that for me to keep milking cows, it wasn’t going to be there and we had to move…get a farm that was secure, rain fed, didn’t rely on irrigation, and nine years out of ten…going to have a good season…pretty much the criteria. (Dairy farmer, Vic.)*

##### Managing farm efficiency

This subcategory of ‘Managing farm efficiency’ conveys that farmers are managing the tactics of operational routines that enables the efficient functioning of adaptation processes.

Farmers identified that learning and problem solving were essential management processes for integrating farm ‘makeovers’ into the system throughout multiple cycles of adaptation:

*The integration of the different system of grazing management was an on-going problem solving and adjustment over many decision cycles… finding the stocking rate that matched the carrying capacity, and livestock type to match the plant type [C3, C4], and discovering the optimum mobs size, plant leaf area as a signal to move on and best pasture rest periods. (Sheep cattle farmer, SW NSW)*

Interviewees described learning and problem solving processes of identifying the weakest point in the system that needed fixing.

*We had to find this weakest link and you work on your weakest link. So if your weakest link is no grass or no water or not enough fences…your weakest link goes higher up the scale…process of elimination…we went out and learnt about it…we made a plan. (Cattle farmer, SE NSW)*.

Interviewees decribed the value of collborative learning and problem solving processes. Consequently, complex biota processes were explained with application to other crops:

*Working with NSW Ag senior soil person and a PhD ….[explain sequeration processes]…it’s that carbon and nitrogen ratio, and activating microbes.. a symbiotic relationship, the plant takes it in carbon through the leave, it takes it down and changes carbon nutrients …..exudates from the roots of the sugar cane…works for soybeans….sprayed the soybeans with another product..it doubled the production of beans. (Sugar-cane farmer, N NSW)*

#### Building resource capability

##### Developing capital resources

This subcategory category of ‘Building resource capability’ conveys that farmers are growing the scale, capability, and resilience of the farm through the agency of adopting *better* and *different* forms of farm resources to match the adaptive demands of the system.

Interviewees described the strategic value of growing and looking after property resources. In response, property signified wealth, enabled economies of business scale, and was buffer to better accommodate the adaptive demands of the changing climate:

*Our biggest capital investment that we have is our land…need to critically look after our land…our biggest risk. If we don’t look after our land, it’s no longer the income producing asset once it was, we’ve let us and our business down. (Sheep-cattle farmer, SW NSW)*.

Interviewees described the strategic development of *different* infrastructure resources to minimise risks of the rising sea level and intense rainfall episodes. Consequently, the landscape was protected, and *better* growing conditions were created:

*We’d laser levelled the whole farm for drainage…[increasing drainage pumping capacity.. this one we’ll be able to deal with four inches 150* *mm rainfall per day … pump in twenty-four hours, at the moment its taking a couple of days. (Sugar cane farmer, NNSW)*

##### Sustaining operating resources

Interviewees identified the adaptive response of optimising equipment capability and scale. Consequently, operational effectiveness and timeliness accommodated the adaptive demands of the changing climate:

*Recently got into precision ag….they develop a prescription for us…their IT bloke..gets the information from our agronomist…do comprehensive soil tests of each zone…paddock is divided into zones….works out what fertilizer rate goes on different zones, so the fertilizer rate changes….variable rate fertilizer… all done automatically…five, six years now. (Cropping-sheep farmer, SE NSW)*

Interviewees described the adaptive response of creating a different flock genotype. In response, livestock potential and scale were matched to the adaptive demands of the changing climate, that led to enhanced productivity and operational effectiveness:

*Started to change them into dual-purpose animal… lifted weaning percentage from 90% to 130%. …17 micron as lambs, 20 micron as adults, fine wool merinos, .. twinning conception to round 65, 70%… joined 400 ewe lambs at six and a half months old… two thirds of those have conceived and 20% have got twins…come from, 75% lambing, wrinkly getting fly struck classic merinos to what we’ve got now, shorn twice a year.. it’s quite, quite amazing… a huge turnaround. (Sheep farmer, Riverina NSW)*

##### Developing human capital

Interviewees identified the importance of developing the resilience of human capital. They described the underdevelopment of metal resilience. Consequently, insufficient resilience meant that farmers were underprepared to handle the on-going stressors and challenges of farm adversities:

*So how do you define resilience…..I’d argue [many farmers] are still leaving something on the table, in terms of our ability…to be socially strong in the face of adversity… the game that we’re involved in…an incredibly difficult one… we face stresses all the time…how do we handle that? …really important part of what we do…mental resilience in the face of adversity, is a thing that we don’t necessarily handle very well…all at different stages with that process. (Grain farmer, Mid-North SA)*

##### Managing financial resources

This subcategory conveys that farmers are managing the ability of the farm business to sustain viability and continuity despite adaptive demand variability.

Interviewees identified differing forms of financial reserves to buffer from variability in the changing climate. Farmers descibed the use of equity to fund drought induced revenue short-falls:

*‘We have the market rate facility… gives us 18 months worth of funding … try to remain flexible within our program…sell off some stock if you think you need to, and know which stock that you would need to sell’ (Cattle farmer, NE Vic.)*

Other interviewees created farm management deposits as an adaptive financial response. Consequently, deposits were withdrawn to buffer short-falls in revenue:

*We’ve got a bit of money invested with a financial advisor…instead of paying tax, you put the money into FMD [farm management deposits]… and I’ll pull some of that out this year because production was a bit low. (Sugar cane farmer, NE NSW)*

Interviewees identified diverse approaches to financing growth in farm capabilities. They described the reinvestment of profits to develop future productivity and reduce risk arising from the adaptive demands of the changing climate:

*After we had a really good year…we did really well. I looked at doing a FMD [farm management deposit]…decided to invest [surplus revenue] back into our pastures… figured if I could grow more grass, and improve my soil nutrient levels…would pay me back over a few years. So that was my choice. (Cattle farmer, NE Vic.)*

#### Transactional map of resilient farm continuity

**Fig. 1:**
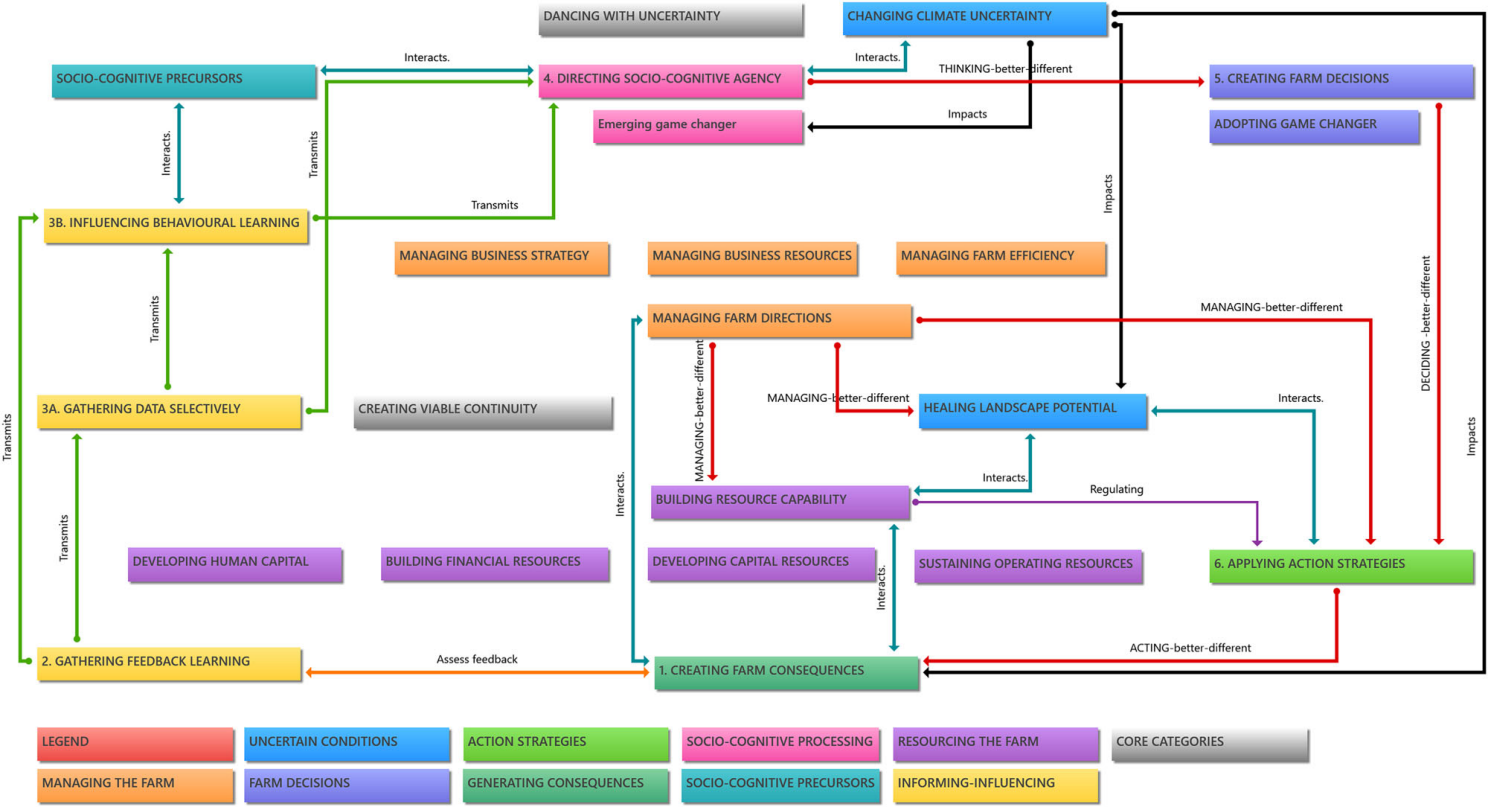
Transactional map of resilient farm continuity. The figure represents the resilience processes of managing farm resources. The transactional map shows the processes and pathways of the resilience and adaptation cycle (steps numbered 1–6). The cycle starts with a farm being impacted by the changing climate that (1) create farm consequences. This is followed the adaptation response sequence of (2) gathering feedback and learning, then (3) gathering intelligence or influencing behaviour. This leads to (4) farmer agency that allows socio-cognitive processing, then (5) to farm decisions, and then (6) applying action strategies that generate further consequences (1), and ongoing cycles of corrective processes. The map focuses on the regulating and transmitting processes between the management and resource elements in the cycle. The map accommodates reactive and proactive forms of adaptation and steps of *better* and *different* forms of thinking, deciding, acting and managing as dual learning pathways (red), i.e. transformative and incremental

Analysis developed the transactional map illustrated in Fig. 1 to represent how resilience processes of managing farm resources and the adaptation cycle (steps 1–6) processes work together. The core category of ‘Creating viable continuity’ suggests that farmers stay in business overtime through the astute management of the adaptation cycle, building human and landscape capital, and financial resource accumulation. In the map, resources act as capital (i.e., landscape, human, economic), with beneficial or adverse consequences of the adaptation cycle adding (e.g., profits, property acquisition) or withdrawing (e.g., income and property losses) from the resources. The points of management leverage in the map are business modelling, corrective management systems, farm problem solving, healing the landscape, and ‘farm make-overs’ for strengthening landscape, social, and productive performance, and continuity. Furthermore, the map shows that certain events and learning experiences (i.e., ‘emerging game changer’) are the catalysts to the sequence of socio-cognitive processing, decision-making (i.e., ‘adopting game changers’), and undertaking adaptive farm make-overs (i.e., innovations) in functions within the farming system, structures and processes.

### Process 3: Adaptation Pathways Processes

Analysis developed a cyclic map of adaptation processes that accounts for key elements and pathway processes of *better* and *different*. The elements of the map, shown in Table 4 represent casual, situational and intervening conditions, action strategies and consequence categories.

**Table 4:** Adaptation pathway connected elements

Conditions		Adaptation pathway processes	
Casual	Changing climate uncertainty	Pathway categories	Creating growing potential
	Growing season potential		Gathering feedback learning
	Healing landscape potential		Gathering intel selectively
Contextual	Shaping uncertain macro-risk		Directing socio-cognitive agency
			Creating farm decisions
		Action-strategies	Applying action strategies
Intervening	Managing business directions	Consequences	Creating farm consequences
	Building resource capability	Core categories	Dancing with uncertainty
	Collaborating business team		Creating viable continuity
	Creating self-determined autonomy		

#### Pathway processes of *better* and *different*

The map found that incremental (i.e., *better*) and transformative (i.e., *different*) adaptation outcomes are incubated at the thinking stage with a sequence of deciding and acting steps. Analysis found six adaptation pathway processes of *better* and *different*: thinking-deciding, deciding-acting, acting-consequences, managing-strategies, managing-landscape, and managing resources.

##### Thinking-deciding

The process of *better* thinking incorporated questioning assumptions that result in having more control in decisions:

*Let’s keep making decisions where we have got control and let’s not worry about things we don’t have control over. But lets keep making sure we understand and make decisions, good decisions, where possible around the areas that we can control. (Sheep-cattle farmer, SW NSW)*.

Interviewees identified that conceiving ideas *differently* involved a different set of world-views about managing the farm system:

*If we want genuine biodiversity, we’ve got to put in place, a diversity of management that might be a diversity of species of animals, a diversity of classes of animals and a diversity of grazing times of grazing intensity. (Sheep-cattle farmer, SW NSW*).

##### Deciding-acting

The process of *better* decision making involved actions that optimised business outcomes:

*You’ve got to make sure you make decisions that don’t have too much of a negative impact on your business… you’ve got to keep that intact… if you can articulate …we’re gonna be selling down stock and you might have a stock agent saying it’s the wrong time to sell…. if you can adequately explain how you arrived at the decision, why you arrived at the decision. (Sheep-cattle farmer, SW NSW*).

Interviewees identified deciding *differently* involved a different set of world-views and structural changes to the farm management system:

*Then this is a big deal, decided …not putting any crop in, like this is mad because we were getting rain…normal autumn… I didn’t even sow anything…I just parked the machinery in the shed…and that year I think I might one of the best, like profit margins, I’d ever made. (Riverina NSW sheep farmer)*

##### Acting-consequences

Interviewees identified that transformative consequences were the outcome of *different* action strategies:

*Work I’ve been doing … for the last 20 years..been able to build the to soil carbon by 3%… capturing around nine tons per hectare per year…spray [crop residue] with five kilograms of urea after harvest… changes the carbon to nitrogen balance…but not to stop the microbes working… if you don’t have a microbial population, you don’t grow crops. (Sugar-cane farmer, N NSW)*

##### Managing-strategies

Interviewees identified integrating *better* and *different* forms of management and the selective application of strategies:

*Able to communicate, monitor, this is going this direction,…how do we turn it around if it is going in the wrong direction. What do we need to do? …so all those questions, normally a diversity of management can resolve a lot of those issues. In any given landscape, there’s areas that are doing well and areas that are doing poorly…give it a diversity of management and over time you cater for a diversity of soil types, landscape types. (Sheep-cattle farmer, SW NSW)*.

##### Managing-landscape

Interviewees identified that a *different* landscape management regime restored healthy functioning in the landscape:

*We’ve got 50 tonne of sulphuric acid per hectare oxidized in the landscape…We’re keeping acid down… it’s an ecosystem service…[developed drainage-land management system]…something we had to do to stay in business…now accepted as world’s best practice for growing sugar cane in acid sulphate soils. (Sugar-cane farmer, N NSW)*

##### Managing-resources

Interviewees identified that *different* resource management produced structural changes to the farm management system:

*Because I’ve got the pumps, my average was higher in the wet years than anybody else…..it was probably 40 [district average], I was 60, at the moment its 115…[cane yield. (Sugar-cane farmer, N NSW)*.

The *transformation* of drainage management and pumping resources boosted crop yields and protected the viability of the fragile landscape.

#### Transactional map of cyclic adaptation

**Fig. 2:**
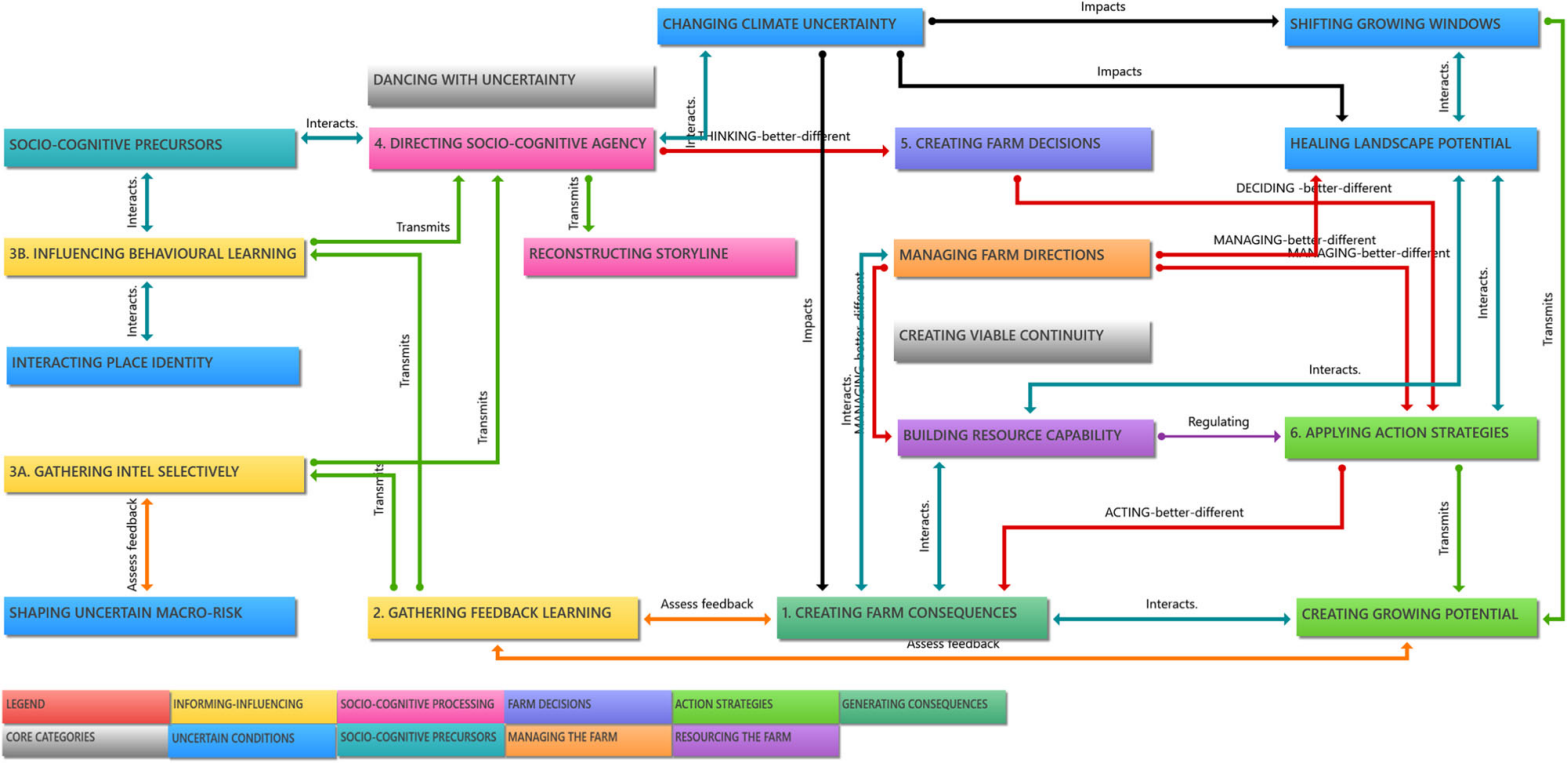
Transactional map of cyclic adaptation. The transactional map shows the processes and pathways of the adaptation cycle (steps numbered 1–6). The cycle starts with a farm being impacted by the changing climate that create (1) farm consequences. This is followed the adaptation response sequence - (2) gathering feedback and learning, then (3) gathering intelligence or influencing behaviour. This leads to (4) farmer agency that allows socio-cognitive processing, then (5) to farm decisions, and then (6) applying action strategies that generate further consequences (1), and ongoing cycles of corrective processes. The process is regulated by intervening management and resource conditions. This map accommodates reactive and proactive forms of adaptation and steps of *better* and *different* forms thinking, deciding, acting and managing as with dual learning pathways (red), incremental and transformational

**Fig. 3:**
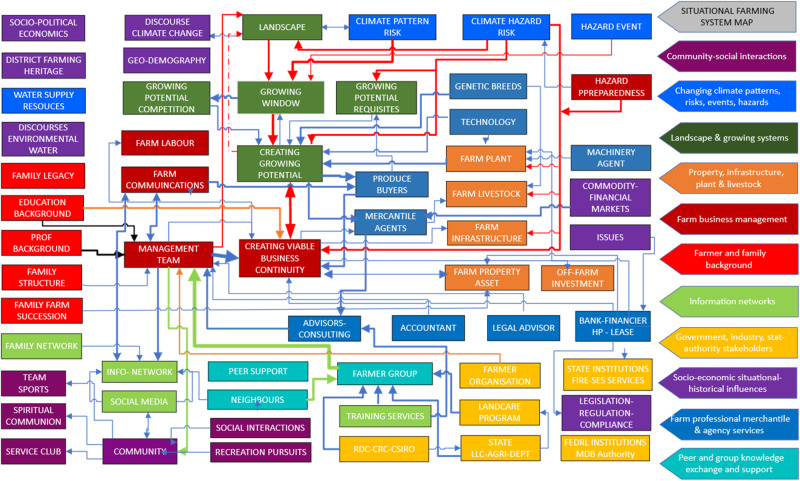
Situational farming systems map. The situational map represents the of the processes and interactions of the biophysical and social ecological elements within the farming system. The map shows the relationships between the elements of farm business, growing system, climate, and actors, actants and discourse within the system

The step-wise process and conditions that influence adaptation are illustrated in Fig. 2. The cycle has six transmission steps that are regulated by intervening conditions. The cycle has multi-level applications at the personal, family, community, and farm business and production level. The cycle starts with a farm being impacted by the changing climate that create (1) farm consequences (crop losses), and the subsequent growing windows, growing season and landscape potential. Subsequently, adaptation cycles produce beneficial consequences (e.g., profits, well-being), and improve farm structures and functions that prepare the farm for future risk. Farmers learn by assessing and gathering feedback (2) throughout the cyclic process. Primary learning from feedback is derived from assessing impacts and anticipating future effects of climate change. Feedback learning is integrated into all the intelligence that farmers selectively gather (3). Farmers seek and are presented with multiple sources of farm data and external sources of information and influence (e.g., peers, groups, advisors, media). Then the socio-cognitive processing occurs (4), by interpreting the intelligence and level of risk, and then selectively thinking about adaptive solutions to problems arising from the original consequences. The efficacy of mental processing depends on influences of socio-cognitive factors.

The decision step (5) is a goal-directed form of mentally processing options, choices, and strategies. The goal is to minimise ‘states of transient dysfunction’ (Norris et al. 2008) and adapt to the changed environment. Decision options are formed and selected. Strategies to achieve goals are informed and influenced by farmer preferences and sources of intelligence. The application of action strategies (4) gives the decision goals tangible form. Action strategies and the growing windows determine the growing potential. That in turn produces consequences. The intervening conditions of management and farm resources regulate actions strategies and growing potential and landscape potential.

## Discussion

The study explored how processes of resilience and adaptation are managed as preparedness and learning responses to climate change in an understudied cohort of farmers. A critical finding was that management, resources, and behavioural learning regulated resilience and adaptation as integrated and self-righting processes, in response to anticipated and actual stressors of climate change.

### Mapping Resilient Farm Continuity

The map of farm resilience identified that the management of resource capabilities and sufficiency, and the corrective actions of adaptation cycles were integrated self-righting processes that ensured farm viability and continuity. These findings extend the Paton et al. (2014) and Norris et al. (2008) explanation of resilience processes by explaining interactions with cyclic adaptation and management processes. At the business level, the management of innovative business models were found to strategically position the farm system while capturing the benefits and minimising losses of evolving climatic conditions. These business models and plans were identified as strategic precursors for transforming the functioning and direction of business and production systems. This is similar to the suggestions of Robertson and Murray-Prior (2016), that farm viability was better achieved by transforming the business. Similarly, Kingwell et al. (2020), suggested that the management of business strategy was vital for sustaining profitable farming systems. This study furthers these claims by adding knowledge of the transformative and management pathway processes of building the resilience of the farm business and resources, needed for stronger farm profits and viability.

The farm system was continually repositioned in step with the changing climate as core resilience and adaptive management strategy to sustain control and business continuity. This finding is consistent with the Wise et al. (2014) principle of ‘adaptive pathways’. Furthermore, this farm cohort developed adaptive management systems (i.e., regimes) and integrated these with the development of business and farming practice transformation. These finding add to the Pahl-Wostl (2006) explanation of ‘learning to manage’ by explaining how farmers were learning and integrating intel from multiple feedback loops throughout short (i.e., tactical) and longer (i.e., strategic) cycles of adaptation. These farmers prioritised the management of natural resources (e.g., soil, water), whilst weighing competing family and economic interests of farm production. These finding are similar to (Everest 2020), that indicated the importance of managing the soil, water, and agroforestry natural resources as adaptation responses to climate change.

The findings suggest that the managed accrual of property and financial resources and development of farm resources (e.g., landscape, people, infrastructure, plant, livestock), strengthened farm resilience and assured viability and business continuity. This is consistent with the Norris et al. (2008) concept of transient dysfunction that robust resource sufficiency (i.e., productive scale, wealth) minimised disruptive stressor consequences. The findings highlight that farmers managed portfolios of insurance and self-managed strategies to reduce the financial risk of climate uncertainty. They transferred hazard risks to insurers for fire and hail. According to Khuu and Juerg Weber (2013), there are limited multi-peril tools for transferring production risks of climate change in Australia. Farmers self-managed downturns in revenue with buffer funds, used short term debt by drawing on equity in properties, and developed property portfolios and/or farm productivity with surplus revenue. These self-reliant strategies for managing financial disruptions of climate change will increasingly be essential for ensuring business continuity.

The findings suggest that farm systems and resources (e.g., landscape, people, infrastructure, plant, livestock) were transformed and improved as preparedness and reactive responses to anticipated and actual climate change stressors. These farm developments (i.e., make-overs) followed a sequence of catalyst events, change incubation, and the integration of adaptive initiatives into the farm system that were regulated by adaptive management. There was a paucity of CCA literature on farmer processes of integrating adaptive initiatives into farming systems. Farm makeovers occurred by integrating multiple domains of self, landscape functioning, infrastructure, equipment technology, and managerial and practice strategies. Adverse hazard and financial conditions often triggered reactive responses, resulting in evolving and larger scale projects of redeveloping the farm. Preparedness responses ranged from one off projects to serial projects that were designed to accommodate future adaptive demands. The catalyst of these responses was autonomous agency (e.g., values), sources of influence (e.g., trusted advisor, peers, groups), and experiential learning and knowledge exchange (e.g., groups).

The acknowledgement of insufficient attention given to developing the resilience of human capital in the farmer sector was a concerning finding. Several farmer studies validate problems of stress and distress arising from climate change (Fleming et al. 2015; Hogan et al. 2013; Wheeler et al. 2018). The findings that farmers were less inclined to prioritise the importance of building psychological capabilities compared with farm productivity represents a systemic vulnerability. This has the potential to amplify as the frequencies and severity of adverse climate events accelerates. Yet, research programs aimed at building the resilience in the triad of people, landscapes and businesses were underrepresented in agricultural literature.

### Mapping Adaptation Pathways Processes

The findings critically identify a comprehensive map of adaptation as a cyclic learning process with a series of incremental and transformative pathway steps (i.e., *better* and *different*). These dual pathways of *better* and *different* were identified as thinking, deciding, acting, and managing strategies, landscape and resources. This transaction map elaborates those developed by climate science (e.g., dual pathway cycle (Park et al. 2012), pathways of decision cycles (Wise et al. 2014)), and environmental science frameworks (e.g., HES (Scholz et al. 2011a)). Firstly, the transaction map illustrates the conditions that interact with the cyclic processes. These conditions include casual (e.g., growing windows, landscape potential, socio-ecological influence), situational (e.g., macro-risk uncertainty), regulating (e.g., property and financial resources), and socio-cognitive precursors (e.g., values, education) that were not specifically identified in prior models. The findings of Everest (2020) identified socio-economic factors, such as land size and education, that had similarity as the influential contributors to the adaptation responses in this cohort of farmers. Secondly, the socio-cognitive agency and consequences variables, and the regulating processes of feedback learning were important inclusions in the map.

The transactional adaptation process accommodates reactive and preparedness responses across multiple scales (i.e., individual, business, community) resulting from stressors and changes in the external environment. Therefore, the cyclic process accounts for stressor induced states of transient dysfunction and resilience processes as described by Norris et al. (2008). The adaptive cycles of problem solving and corrective action, identified by these farmers was consistent with the pathways model of action learning cycles conceptualised by Wise et al. (2014). In this study, farmers thought outside the box and created *different* farming systems by synthesising principles and techniques. This transactional map has diagnostic utility for identifying variables at various levels of the system, that have the most potency for strengthening responses at each step of the adaptation cycle. These findings make an important contribution to the broader need for models that articulate farmer decision-making, that are integral in ensuring adaptation to agricultural challenges and opportunities related to climate change (Adelhart Toorop et al., 2020). Further research is needed to identify various factors that have the most efficacy as levers in strengthening various stages and levels of adaptive responses.

### Framing Effects of Climate Change

The findings uniquely identify that the changing climate was framed as effects on growing windows and the growth potential of plants and animals. The language frames of growing windows were used to demarcate temporal and climate limits of active plant growth (e.g., crops, pastures). Farmers also framed growth potential to account for beneficial and adverse effects of climate change (e.g., floods, fire, hail, heat stress), growth competition factors (e.g., weeds, pathogen), and growth enhancing factors (e.g., fertilisers). The force field approach has value for identifying which of these beneficial and adverse variables have efficacy for improving growth potential (Burnes and Cooke 2012). This cohort of farmers experienced instability in growing season patterns of rainfall and temperature, whilst climate variability extremes were amplified. The frame of climate variability had continued application to these climate change phenomena due the use of learnt interpretive frames. Farmers acknowledged that these chronic phenomena had little hope of restoration, and likely to last indefinitely. However, disruptions faced by well-prepared farmers due to climate change consequences were fewer, they adapted more readily, and sustained viable businesses and continuity of farm systems.

### Implications of These Findings

The findings of the current study have several implications for theory, management, and policy. The study adds to theorical knowledge by developing conceptual maps that capture the interdependent processes of resilience and adaptation. Resilience arises from the management of resources interacting with cyclic processes of transformation, learning and corrective action. The adaptation map illustrates the processes of thinking, deciding, acting and managing that accounts for transformational and incremental forms of adaptation. Furthermore, the map accounts for the precursors of adaptation and interacting factors that transmit and regulate the processes.

The study adds to the knowledge of farm business and management systems. We identified that farm businesses need to be strategically positioned and managed to keep in step with adaptive demands of evolving climate change. The strategic design and management of business models were precursors to transforming business and production systems, and pivotal to capturing the benefits of, and minimising losses of, evolving climatic conditions. The strategic management and viability of all balance sheet assets, including financial, human and natural resources, were markers of resilient farming systems. Farm resilience is crucial for the adaptive preparedness required for counteracting the uncertainty of climate disruptions, through re-positioning of farm businesses. Furthermore, transformative forms of adaptation were often triggered by disruption, implying that salient threats to business continuity motivated adaptive behaviours, in this particular cohort of innovative farmers. Farm make-overs occurred as evolving, scalable, developmental projects that restored and protected landscape functions, improved productivity, and improved management practices. These projects were mediated by experiential learning and active change management, regulated by self and collective efficacy within supportive cultural environments. The study further adds to knowledge that the changing climate can be framed as effects on growing windows and the growth potential of plants and animals. These have management applications as indictors of climate change by tracking changes in growing windows and evaluating changes to growth potential.

Based on the findings, it is recommended that policy makers place greater emphasis on creating policy frameworks that foster adaptation and preparedness. These should emphasise specific programs that build strategic business and financial management capabilities, and psychological resilience.

## Limitations

The maps do not set out to define a generalised set of outcomes produced by the composite model, rather forms a starting point from which to direct future research. The various types of farms that are represented by the data have adapted and developed resilience and preparedness, which attests to their viable continuity. The farm sectors represented by farmers in the study may not reflect other farm sectors and the interactions between management, resources and situational context.

## Conclusions

This study provides comprehensive maps of managing resilience and adaptation processes, by this specific cohort of farmers. These maps provide important detail about the pathways and variables that are key to the self-righting processes of preparedness and resilience. The findings emphasise that the viability of farm businesses and continuity of the farming system depend on thinking and managing differently. Although previous studies have suggested some of these key adaptation mechanisms, such as the dual pathway model, our map provides a range of contextual conditions and potential transmission and regulating variables. These elements offer policy makers useful information about points of leverage to consider when working with farmers in co-creating programs aimed at broadening the scope of adoption and rate of farmer adaptation.

## Appendix

Fig. 3
